# Cost-minimizing team hires with participation constraint

**DOI:** 10.1371/journal.pone.0201596

**Published:** 2018-08-28

**Authors:** Heli Sun, Jianbin Huang, Ke Liu, Mengjie Wan, Yu Zhou, Chen Cao, Xiaolin Jia, Liang He

**Affiliations:** 1 Department of Computer Science and Technology, Xi’an Jiaotong University, Xi’an, Shaanxi, China; 2 School of Software, Xidian University, Xi’an, Shaanxi, China; University of Texas at San Antonio, UNITED STATES

## Abstract

Team formation, which aims to form a team to complete a given task by covering its required skills, furnishes a natural way to help organizers complete projects effectively. In this work, we propose a new team hiring problem. Given a set of projects P with required skills, and a pool of experts X, each of which has his own skillset, compensation demand and participation constraint (i.e., the maximum number of projects the expert can participate in simultaneously), we seek to hire a team of participation-constrained experts T⊆X to complete all the projects so that the overall compensation is minimized. We refer to this as the participation constrained team hire problem. To the best of our knowledge, this is the first work to investigate the problem. We also study a special case of the problem, where the number of projects is within the participation constraint of each expert and design an exact algorithm for it. Since participation constrained team hire problem is proven to be NP-hard, we design three novel efficient approximate algorithms as its solution, each of which focuses on a particular perspective of the problem. We perform extensive experimental studies, on both synthetic and real datasets, to evaluate the performance of our algorithms. Experimental results show that our exact algorithm far surpasses the brute-force solutions and works well in practice. Besides, the three algorithms behave differently when distinct facets of the problem are involved.

## Introduction

A successful recruitment process or project bidding process devotes to hiring a set of experts from a batch of candidates that satisfy the requirements of specific projects. From the perspective of managers how to form a cost-efficient team to accomplish specific projects is one of the most essential issue. In most cases, the specific properties of experts, such as professional expertise [1], work time, the maximum workload [2] and leader evaluation and team cohesiveness [3] vary among different individuals. Such factors may affect the amount of projects they want to engage in simultaneously. Therefore, an efficient hiring process should take both the cost and the ability of experts into consideration.

Assume such a scenario, a software company wants to build a team of engineers to develop a number of mobile applications, supplying programmers, system architects, product managers, UI designers and technical advisers separately for each application. In this setting, creating a skilled and cost-effective team for the projects is desired for managers. We can easily come up with a solution that each position just needs to find one appropriate expert to guarantee the completion of it. However, it seems unreasonable from the perspective of experts that no distinction is made between the numbers of projects each member can enter. Due to the heavy workload inherent in coding, programmers are more inclined to engage in only one project, while technical advisers may take an active part in multiple projects at the same time depending on entailed by work. Thus, the number of projects each expert participates in, i.e., the participation constraint [4], varies among different individuals. Effective team hiring process should take this trait into consideration whose significance has been proven in many real-world scenarios.

However, team hiring is not limited to the domain of software development only. In the setting of film industry, social-bookmarking, academic cooperation and crowdsourcing, teams are fundamental to these collaborative scenarios, where both the cost of a team and the ability of experts are essential and should be considered [2, 5, 6]. In addition, there are some other works related to team hiring problem such as top-k team formation [7], analytical team formation [8] and social event organization [9–12] and so on. Overall, the problem of hiring a team of experts for collaborative projects has extensive real-world applications and is an important problem to study.

We illustrate aforementioned characteristics more concretely through the following simple example. We assume there is a manager who wants to build a team of experts to perform the following projects: P={1,2,3,4}, with required skills shown in Table 1. Also assume there are eight experts, X={A,B,C,D,E,F,G,H}, equipped with the skills, cost and participation constraint denoted by p-constraint listed in Table 2.

**Table 1:** Skills of projects.

projectid	1	2	3	4
skills	{a,b,c,d,e}	{a,c,d,e,f}	{b,c,f,g}	{a,d,e,g}

**Table 2:** Skills, cost and participation constraint of experts.

expertid	A	B	C	D	E	F	G	H
skills	{a,b}	{d,e}	{b,c,d}	{a,f,g}	{c,f}	{d,e,g}	{b,c,f}	{a,b,e}
cost	5	10	3	6	4	5	3	4
p-constraint	2	4	2	3	2	3	2	2

**Fig 1:**
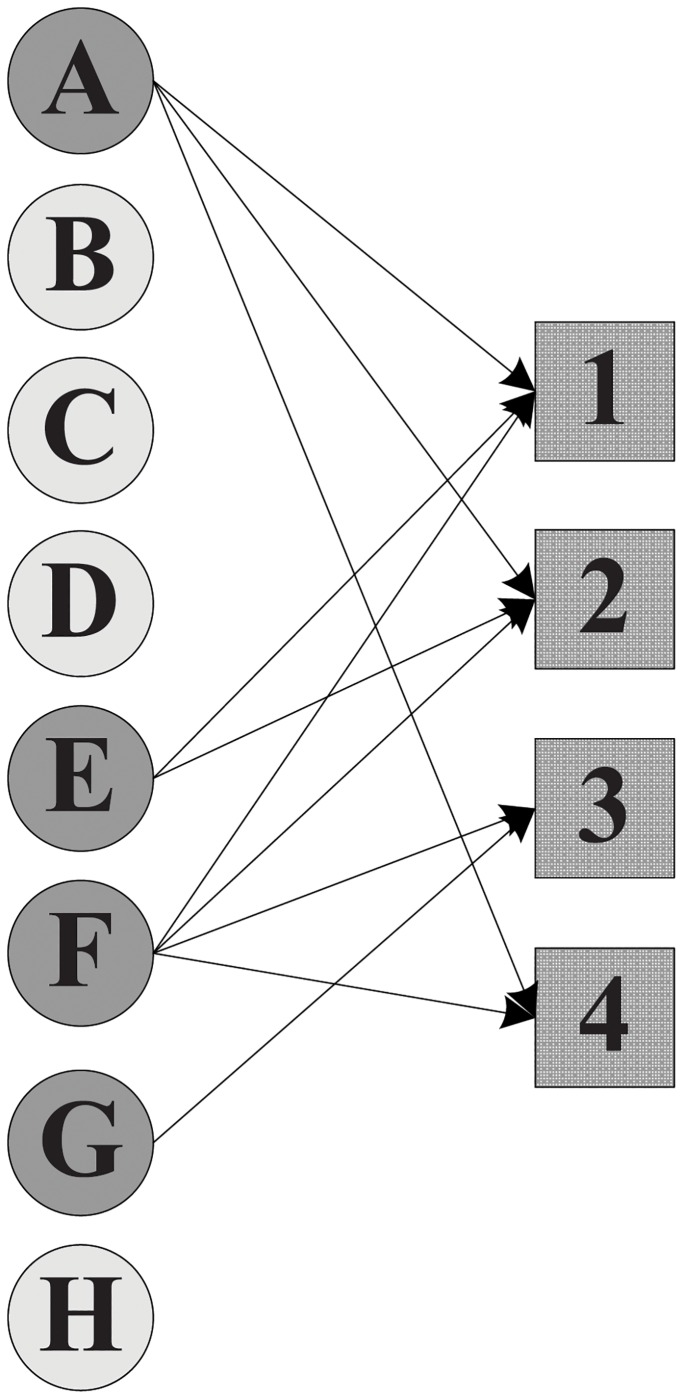
An assignment scheme without participation constraint.

**Fig 2:**
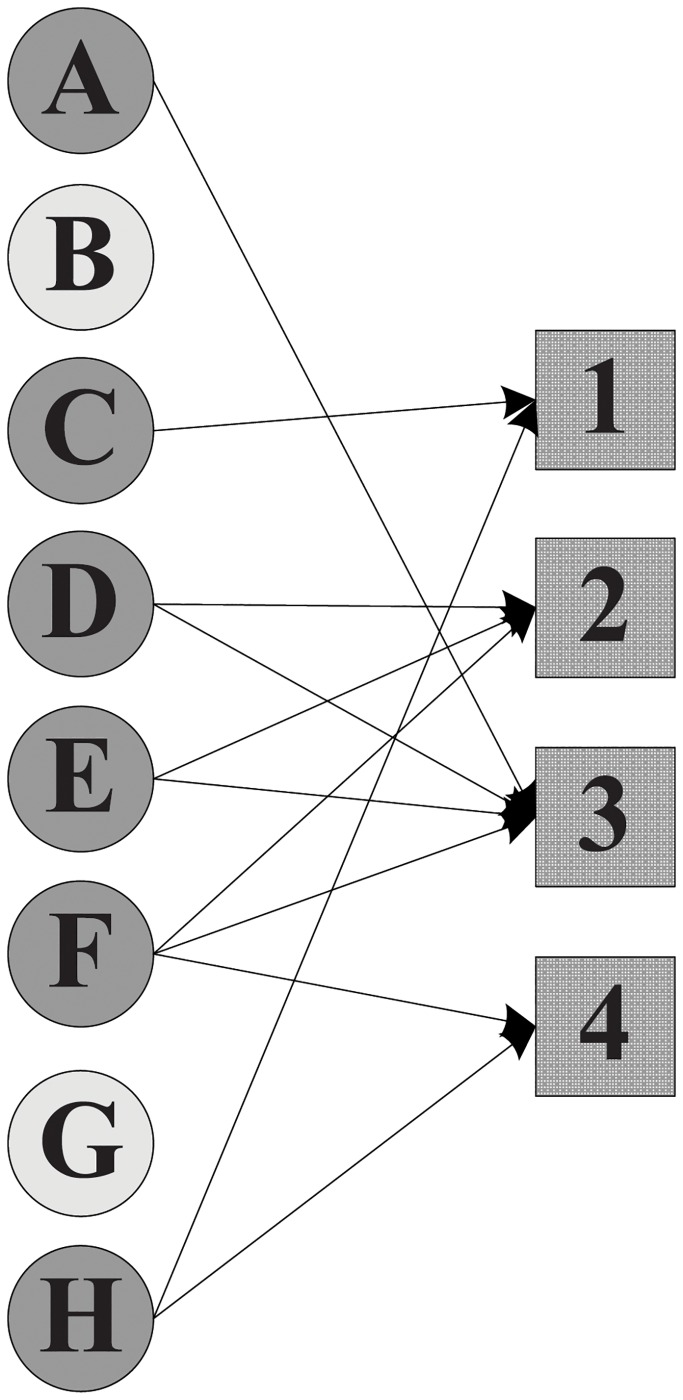
The cost of an assignment scheme with participation constraint is 27.

**Fig 3:**
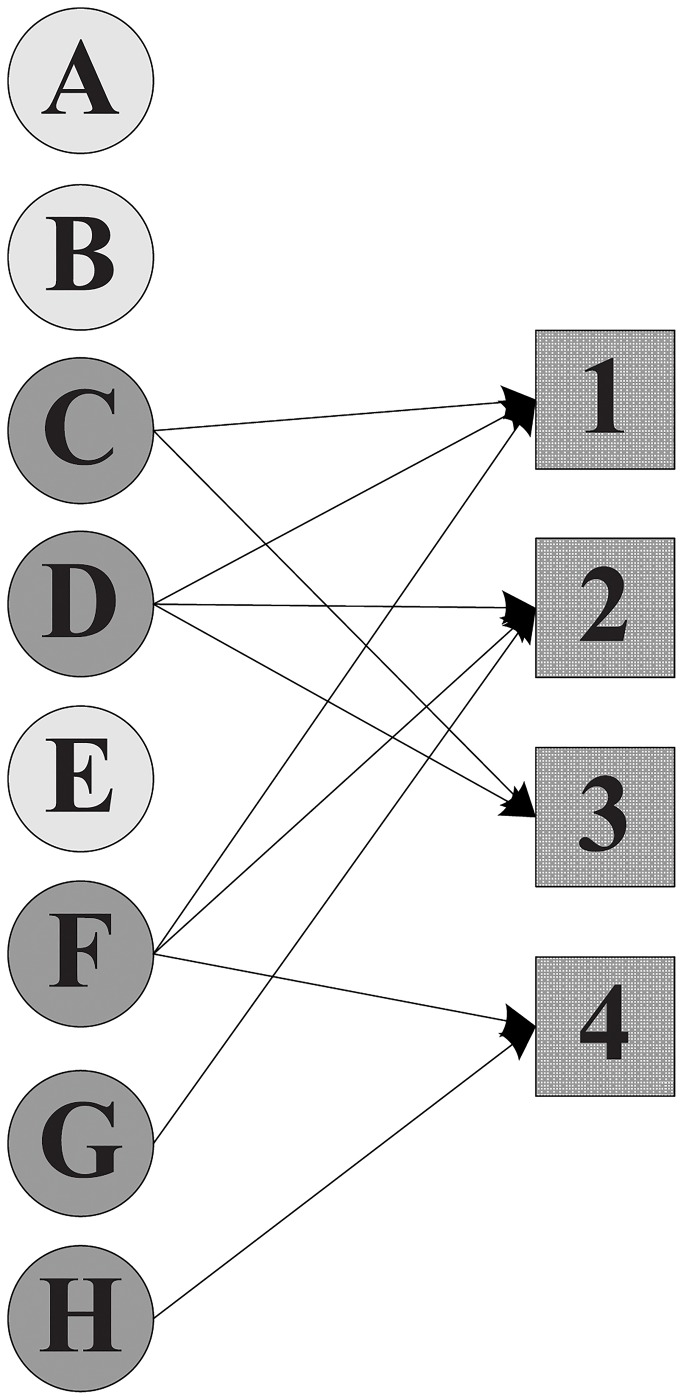
The cost of an assignment scheme with participation constraint is 21.

Without considering the participation constraint of each expert, the manager can select either *X* = {*A*, *E*, *F*, *G*}, *X*′ = {*A*, *C*, *D*, *E*, *F*, *H*} or *X*″ = {*C*, *D*, *F*, *G*, *H*}, since all these teams can collectively cover the required skills of the projects. Fig 1 depicts the assignment scheme without participation constraint, where apparently expert *F* joins four projects in parallel while expert *G* enters merely one project. Then, after imposing the participation constraint on project assignment, the resulting assignment schemes are shown in Figs 2 and 3. Furthermore, the comparison of the total cost that 21 incurred by *X*″ is less than 27 by *X*′ suggests that *X*″ = {*C*, *D*, *F*, *G*, *H*} is a superior solution.

Motivated by the above observation, in this paper, we formalize the problem inspired by [13] in the following. Assume a pool of *n* experts X, where each expert $x_{i}\in X$ possesses a set of skills *s*(*x*_*i*_). Additionally, we assume a set of *m* projects P, for each project $p_{j}\in P$, *s*(*p*_*j*_) is composed of the skills required to complete the project. Finally, every expert is associated with a cost function *c*(*x*_*i*_) which corresponds to *x*_*i*_’s compensation and a participation constraint function *w*(*x*_*i*_) which represents the number of projects expert *x*_*i*_ can engage in at most during the same period of time. Our goal is to form a team of participation-constrained experts T⊆X to complete all given projects such that the total cost is minimized. We assume that T can complete all the projects P only if for each skill required by project $p_{j}\in P$, there exists at least one member in T who can cover it. To be clear, for each expert we only consider his skills which are required by projects. We call this problem the participation constrained team hire problem. In addition to the fundamental problem, we also tackle a special case of participation constrained team hire problem where the number of projects is within the participation constraint of each expert, such that we can ignore the participation constraint of experts. We dub the preceding special case the participation free team hire problem. Although both the participation constrained team hire problem and the participation constrained team formation problem proposed in [4] consider the participation constraint, they have different objectives.

In this paper we proposed the participation constrained team hire problem and a special case of it, and our major contributions are summarized as follows:

1. To the best of our knowledge, we are the first to define and study the participation constrained team hire problem (PCTH). We impose the participation constraint of experts so that no expert is overworked under the resulting assignment scheme. We define a special case of PCTH called participation free team hire problem (PFTH) where the number of projects stays within the participation constraint of each expert.
2. Though we prove that PFTH is NP-hard, two exact algorithms, integer programming and Linking-Pruning Algorithm (LPA), are proposed, which can discover the best team based on well-designed pruning strategies.
3. We show that PCTH is NP-hard to solve, and design three effective algorithms, each of which focuses on a particular dimension of the problem. Then, we conduct a careful and detailed set of experiments to evaluate the performance of the proposed algorithms.

The rest of the paper is organized as follows: in Section we review the related work. Section formally defines the PCTH and PFTH respectively, and analyzes the complexity of the two problems. Our exact algorithm for PFTH is described in Section, and in Section we present three algorithms for PCTH. In Section we perform extensive experimental studies for the evaluation of our methods. We conclude the paper in Section.

## Related work

To the best of our knowledge, we are the first to introduce and study PCTH. However, our problem is also related to some well-studied ones. We give an overview of their treatment on this subject below.

(Team Formation Problem) Lappas et al. [14] first introduced team formation in the context of social networks. In recent years, many researchers [15–22] extend this work. All these extended variations except [17] assume the context of a social network and therefore, their formulations and solutions are graph-theoretically based. Our work does not make this assumption and differ markedly from theirs. Anagnostopoulos et al. [17] do not assume a network of experts. In their paper, a collection of projects with different skill requirements arrive one at a time in an online fashion, and for each project coming, they create a team for it. Their goal is to minimize the maximum number of teams that each expert participates in. Obviously, our work diverges considerably from theirs in three aspects. First, our projects are known apriori. Second, we only create a single team for all the projects and do not create teams for each project individually. Third, their optimization aim is to minimize the maximum number of teams that each expert participates in while our goal is to minimize the overall compensation of the team.

(Set Cover Problem) Our work is also related to the Set Cover (SC) problem [23–26], especially the weighted Set Cover (WSC) problem [27–30] and the Set Multicover (SMC) problem [31–34]. In the set cover problem, given a universal set *E* and a set of subsets of it which are called *S*, the goal is to find a minimized collection of sets from *S* such that it covers all the elements in *E*. Weighted Set Cover problem defines a nonnegative weight for each set in *S*, and attempts to minimize the total cost of the found sets. Set Multicover problem is predicated on a multiset *N* instead of the universe *E*. Here a multiset *N* contains a specified number of duplicates of each element *n*_*i*_ ∈ *N*, which is denoted by *b*_*i*_. The objective is to find a minimum cardinality subset such that each element *n*_*i*_ ∈ *N* is covered by *b*_*i*_ times. All the Set Cover problems involve one universal set, However, because of the participation constraint, we are unable to merge the projects together, which implies that there is more than one universal set. This is where the primary distinction between these two problems lies.

(Cluster Hire Problem) Perhaps the closest work to ours is the Cluster Hire problem [13]. Given a set of projects, each project is characterized by the skills that are necessitated for its completion. Additionally, each project is associated with a profit gained upon its completion, and every expert incurs a cost corresponding to his compensation. The goal of ClusterHire is to form a team of experts such that the total cost does not exceed the specified budget and the total profit stemming from the projects accomplished by the team peaks. Differences between Cluster Hire problem and our problem are two-fold. First, our problem takes the participation constraint into consideration which implies our experts are not inexhaustible. But experts in Cluster Hire problem are inexhaustible and an expert can be assigned to an arbitrary number of projects. Second, our problem intends to create a team of experts to handle all the projects while minimizing the overall compensation. However, Cluster Hire aims to form a team of experts whose total salaries stays below the budget while maximizing the overall financial gain. In their work [13], they also consider a variant of ClusterHire. The variant places an upper bound on the number of projects for which an expert can utilize a skill *a*_*k*_. This setting is different from ours since our participation constraint limits the individual rather than their skills. [6] proposed an more effective algorithm for the Cluster Hire problem. [4] imposed a participation constraint on the Cluster Hire Problem, and proposed an effective algorithm for the problem.

(Reviewer Assignment Problem (RAP)) Reviewer Assignment Problem [35–38], which coordinates the assignment of reviewers to papers, also behaves like our problem. However, differences between the two problems are also evident. First, each paper must be reviewed by a fixed number of reviewers in the setting of RAP while in our problem, the size of expert set attached to each project is not rigidly constrained. Second, the skills required by each project in our problem must be totally covered while the topics of each paper are not purported to be completely satisfied in RAP, and in most cases, this is expected. Third, the ultimate objective of our problem is to minimize the overall compensation, however, RAP devotes itself to maximizing the covered topics.

## Problem definition

In this section, we first introduce some concepts we will use to define the problems. Then, we formulate the participation constrained team hire problem and participation free team hire problem respectively, and analyze their corresponding complexity.

### Concepts

We assume there is a set of *k* skills $A=\{a_{1},\ldots ,a_{k}\}$, a set of *m* projects $P=\{p_{1},\ldots ,p_{m}\}$ and a set of *n* experts $X=\{x_{1},\ldots ,x_{n}\}$. Projects P need to be all accomplished and we use a skill function (*s*), such that for each project $p_{j}\in P$, *s*(*p*_*j*_) denotes the set of skills required by *p*_*j*_ for its completion, $s(p_{j})\subseteq A$. Similarly, each expert $x_{i}\in X$ is associated with a set of skills which we also designate it by *s*(*x*_*i*_), $s(x_{i})\subseteq A$. In addition, we have a cost function (*c*) and a participation constraint function (*w*), such that for every $x_{i}\in X$, *c*(*x*_*i*_) gives the cost of hiring *x*_*i*_ and *w*(*x*_*i*_) specifies the maximum number of projects *x*_*i*_ can engage in simultaneously, *w*(*x*_*i*_) ≥ 1.

To complete all the obligatory projects we need to hire a team of experts. Let T⊆X be a team established to cover the requirements of all the projects. T also constitutes a certain skill set, which is computed as the union of the skills of its members. That is, $s(T)=\cup_{x_{i}\in T}s(x_{i})$. After a team of experts T is formed, each project $p_{j}\in P$ can be completed by one of T’s subsets. We define a complete function (*com*), such that for each $p_{j}\in P$, *com*(*p*_*j*_) stands for a subset comprising experts of the formed team which are allocated to *p*_*j*_. Taking Fig 3 as an example, *com*(2) = {*D*, *F*, *G*}, which represents that experts *D*, *F* and *G* are in charge of project 2. Table 3 summarizes the terse notations we described above.

**Table 3:** Notations.

A	Set of skills
P	Set of projects
X	Set of experts
T	A formed team
*k*	Number of skills
*m*	Number of projects
*n*	Number of experts
*s*(*x*_*i*_)	Skills of expert *x*_*i*_
*s*(*p*_*j*_)	Skills of project *p*_*j*_
*c*(*x*_*i*_)	Compensation cost of expert *x*_*i*_
*w*(*x*_*i*_)	Participation constraint of expert *x*_*i*_
*com*(*p*_*j*_)	Subset experts assigned to project *p*_*j*_

For a team of experts T⊆X and a project $p_{j}\in P$, we say that T can cover *p*_*j*_ if T encompasses all the required skills for *p*_*j*_, i.e., $s(p_{j})\subseteq s(T)$. Obviously, the formed team is capable of covering more than one project. Thus, we introduce the coverage of a team T in Definition 1, and a similar notion can be found in ClusterHire [13]. To ensure all the team members are not overworked, we present the feasibility of a team in Definition 2. Additionally, every team incurs certain expenses, hence Definition 3 gives the computed total cost of a team.

Definition 1 (Coverage). Given a set of projects P and a team T, we define the coverage of T to be the set of projects that T can cover. That is,

$$\begin{array}{c} Cov(T)=\{p_{j}|p_{j}\in P\wedge s(p_{j})\subseteq s(T)\}. \end{array}$$ (1)

As illustrated in Fig 3, the coverage of team *X*″ is $Cov(X^{\prime \prime })=\{1,2,3,4\}$.

Definition 2 (Feasible Team). Given a team of experts T⊆X which is formed to handle a set of projects P, we say that T is a feasible team if for each $x_{i}\in T$, the number of projects he participates in is within his participation constraint, i.e., for each $x_{i}\in T$, $\left|\cup_{p_{j}\in P:x_{i}\in com(p_{j})}p_{j}|\leq w(x_{i}\right)$.

In our running example, team *X* shown in Fig 1 can not be a feasible team because expert *F* shares the workload with others in 4 projects in parallel while his given participation constraint *w*(*F*) in Table 2 falls short, i.e., $|\cup_{p_{j}\in P:F\in com(p_{j})}p_{j}|=|\{1,2,3,4\}|=4⩾w\left(F\right)=3$. Apart from *F*, expert *A* whose participation constraint has been violated too also renders team *X* infeasible.

Definition 3 (Team Cost). Given a team of experts T⊆X, we define the cost of the team as c(T), computed by the sum of the costs of its members. That is,

$$\begin{array}{c} c(T)=\sum_{x_{i}\in T}c(x_{i}). \end{array}$$ (2)

As we can see in Table 2, expert *E* is associated with the compensation cost 4, notated *c*(*E*) = 4. Also, we can easily calculate the total cost of the team *X*″ in Fig 3 as *c*(*X*″) = *c*({*C*, *D*, *F*, *G*, *H*}) = 3 + 6 + 5 + 3 + 4 = 21.

### The participation constrained team hire

Having introduced the foregoing preliminaries, we can now formulate the participation constrained team hire problem addressed in this paper as follows:

Problem 4. Given a set of projects P, a set of experts X, we seek to find a team T⊆X, such that

1. Cov(T)=P;
2. T is a feasible team;
3. c(T) is minimized.

We abbreviate the name of the problem to PCTH. By definition, PCTH is a constrained optimization problem. From the computational point of view, we have following results for this problem.

Theorem 1. *The decision version of participation constrained team hire problem is NP-complete*.

Proof. We prove the theorem by a reduction from the SetCover problem. In the classical SetCover problem there is a universe of items *U* and a set of sets *S* = {*S*_1_, *S*_2_, …, *S*_*k*_} such that for every *S*_*i*_ ∈ *S*, *S*_*i*_ ⊆ *U*. Given a constant *K*, the decision version of SetCover problem is whether there exists *S*′ ⊆ *S* such that $\cup_{S_{i}\in S\prime }S_{i}=U$ and |*S*′| ≤ *K*.

Now, we concentrate on a simplified version of the problem which stipulates that experts can participate in all projects without any constraints, i.e., $\forall x_{i}\in X$, *w*(*x*_*i*_) = ∞. Moreover, we specifically consider a special case that P consists solely of a single project and *c*(*x*_*i*_) = 1. In this case, we are only concerned with the amount of experts. Thus, the problem now transforms into finding a feasible assignment *X*′ that minimizes the cost to complete all projects.

Clearly, if we map every set *S*_*i*_ ∈ *S* from SetCover problem onto *s*(*x*_*i*_) of PCTH, the two problems become identical. That is, there exists a solution of cost *K* in the SetCover problem if and only if there exists a solution of cost *K* in PCTH.

Theorem 2. *The participation constrained team hire problem is NP-hard to approximate*.

Proof. The proof of the above theorem leverages the same simplified decision version of PCTH employed in the proof of theorem 1. We create an instance Γ of SetCover and a PCTH instance T based on the simplified decision version. Through our construction, *OPT*_Γ_ = *OPT*_T_, i.e., a feasible solution for instance Γ is identical to the one for instance T.

We now prove this theorem by contradiction. That is, assume that there exists an approximation algorithm Λ with approximation guarantee [39] *α* (also called approximation factor, i.e. the supremum of the fraction of the approximate value to the optimum value for all the problem instances) for this simplified version of our problem. Then, running Λ on T can decide whether a solution comprising *K* experts who manage to perform all the projects of our problem can be discovered. Apparently, algorithm Λ is suitable for instance Γ. However, this deduction flatly contradicts to the previous findings by Lund and Yannakakis who showed that SetCover problem cannot be approximated in polynomial time unless NP has quasi-polynomial time algorithms [23]. Therefore, such an approximation algorithm with approximation guarantee *α* does not exist.

In the definition of PCTH, we focused on minimizing the compensation cost with participation constraint. If the participation constraint was not a concern, our goal would change to find a team T such that Cov(T)=P and c(T) is minimized. Such a problem definition is actually an instance of the classic Weighted Set Cover problem since all the projects can be merged into one whose required skills is the union of its members’. If the merged project asks for a particular skill *a*_*k*_, so will at least one of the projects constituting its combined counterpart. Besides, that *a*_*k*_ is demanded by most projects further aggravates this issue. To put it simply, if an expert who owns the skill *a*_*k*_ is selected to cover the projects, he is very likely to join too many projects and overwork. Our work precisely attacks such a problem. Taking the participation constraint into account, each expert can only be assigned to a limited (and usually very small) number of projects, suggesting that the projects ought not to be merged into one which covers each skill only once.

Alternatively, we may attempt to aggregate the projects into one and convert the skill set of this combined version into a multi-set, so the crux of the issue can be now viewed as a Set Multicover problem. Clearly, the number of duplicates of each skill indicates how many times the skill should be utilized. However, having merged all of them, we are not able to discern which project each skill initially belongs to. Furthermore, even if a particular team may seem fit for the merged project, where each skill required can be covered multiple times, we can hardly add up the statistics of projects that an expert in the team enters in parallel. Consequently, whether the participation constraint is satisfied can not be interpreted from this team formation scheme, once again reminding us that the projects ought not to be integrated into one.

Therefore, the essence of PCTH is the participation constraint and the critical factor of the solution resides in the fact that the projects must be kept separate from one another and can not be merged. Here lies the core difference between previous pertinent work and our problem which is more common in practical applications and more difficult to tackle.

### The participation free team hire

In this section, we present a special case of the participation constrained team hire problem, called participation free team hire, where the participation constraint of each expert exceeds the total quantity of all the projects. Therefore, we can ignore the participation constraint of experts, since even if an expert engages in all the projects, the participation constraint will not be violated. Therefore, given a set of *m* projects $P=\{p_{1},\ldots ,p_{m}\}$, our goal is to hire a team of experts to manage these projects and minimize the overall compensation cost. Formally, the participation free team hire problem (PFTH) can be condensed into the following definition:

Problem 5. Given a set of projects P, a set of experts X, we seek to find a team T⊆X, such that

1. Cov(T)=P;
2. c(T) is minimized.

As has been discussed above, with the participation constraint having no decisive effect on our problem, the candidate projects can be merged into a larger one whose required skills is the union of skills of its members. The following theorem proves the NP-hardness of PFTH.

Theorem 3. *The participation free team hire problem is NP-hard*.

Proof. We will show that the NP-hard Weighted Set Cover problem can be reduced to an instance of PFTH. Given a universe of items *U* and a set of sets *S* = {*S*_1_, *S*_2_, …, *S*_*k*_} such that for every *S*_*i*_ ∈ *S*, *S*_*i*_ ⊆ *U*, where each set *S*_*i*_ is assigned a cost. The Weighted Set Cover problem attempts to find a subset *S*′ ⊆ *S* such that ⋃_*S*_*i*_ ∈ *S*′_ *S*_*i*_ = *U* and the total cost of *S*′ is minimized. Our problem considers a set of projects P, and a set of experts X where each expert $x_{i}\in X$ features a skill set *s*(*x*_*i*_) and a cost *c*(*x*_*i*_). The goal is to work out a combination of experts which can collectively cover the projects and the total cost is minimized. Since projects in PFTH can be merged into one and the skills required by the project is the union of the skills of its members, we can first aggregate the projects into one project *P*′, and then discover a team of experts to handle *P*′ while minimizing the total cost. PFTH is equivalent to the Weighted Set Cover problem if we map every set *S*_*i*_ from the Weighted Set Cover problem onto an expert skill set *s*(*x*_*i*_) of PFTH and similarly map *U* onto the skill set of project *P*′. Thus, the PFTH problem is NP-hard.

The most valuable feature of PFTH rests on a special case where only a single project needs to be completed (i.e., P={p}). This case frequently emerges in practical applications. For instance, a software company is looking for programmers for one cellphone application or the medical personnel want to closely cooperate with their peers and perform an emergency surgery for their patients. These scenarios merely require one project.

## Two exact algorithms for pfth

Here, we introduce two exact algorithms for problem PFTH. In Section, we introduce the linking-pruning algorithm based on the Aprior algorithm. In Section, we introduce the integer programming based algorithm.

### Linking-pruning algorithm

Below, we introduce an exact algorithm for PFTH as a baseline. This algorithm is based on Apriori algorithm [40]. Its time efficiency is comparable to integer programming when the number of skills is relatively small. In real world, experts usually have relative small skill. Obviously, Brute Force Search which enumerates every possible team can be employed to address our problem exactly. However, this solution is very sensitive to the size of expert set and does not scale well since it examines every possible permutation. In this section, we delineate our exact algorithm Linking-Pruning algorithm (LPA) for PFTH. Given $P=\{p_{1},\ldots ,p_{m}\}$, from the problem definition we can merge P into a large project *P*′, and the skills required by *P*′ is the union of skills of its members, i.e., $s\left(P^{\prime }\right)=\cup_{p_{j}\in P}s\left(p_{j}\right)$. To be clear, the algorithm described in this section proceeds to the completion of *P*′. Evidently, if *P*′ is done, so will be all the projects in P.

We adopt the thought of Apriori Algorithm [40] for mining association rules to reduce the search space of our problem. That is, LPA employs an iterative method searching layer by layer to examine all the promising permutations which eventually facilitate identifying the optimal solution. First, given *Eset* to represent an expert set, and *k*-*Eset* for an *Eset* with *k* experts. In each layer, we start with a seed set of *k*-*Eset*s, notated as *L*_*k*_ (*k* ≥ 1), and try to use *L*_*k*_ to generate *L*_*k*+1_. However, the scale of *L*_*k*_ might be large, so the computational cost can be prohibitively high. To compress *L*_*k*_, we scan the whole *L*_*k*_ and determine which of those *k*-*Eset*s in *L*_*k*_ has the potential to be a component of the best team. We then exclude those *k*-*Eset*s which would never contribute to our solution, and obtain the k-candidate expert set $I_{k}^{E}$. Finally, we link $I_{k}^{E}$ with $I_{k}^{E}$ to generate all (*k* + 1)-*Eset*, i.e., *L*_*k*+1_, and then *L*_*k*+1_ becomes the seed set for the next layer. The main process consists of two steps: Linking and Pruning.

Linking. To reduce the search space, $I_{k}^{E}$ instead of *L*_*k*_ is used to generate *L*_*k*+1_, since all *k*-*Eset*s in $I_{k}^{E}$ are potential candidates. We achieve this by linking (notated as ⋈) $I_{k}^{E}$ with $I_{k}^{E}$. What should be clear is that, $\forall i,j\in I_{k}^{E}$, *i* and *j* can be linked only if the newly formed expert set is a (*k* + 1)-*Eset*, i.e., $L_{k+1}=I_{k}^{E}⋈I_{k}^{E}=\left\{i\cup j|i,j\in I_{k}^{E}\wedge \left|i\cup j\right|=k+1\right\}$. The constraint |*i* ∪ *j*| = *k* + 1 stipulates that every generated expert set is a (*k* + 1)-*Eset*.

We now illustrate the linking step with our running example. Let $I_{2}^{E}$ be {{*A*, *G*}, {*C*, *G*}, {*G*, *H*}, {*E*, *F*}}. After the linking step, *L*_3_ will be {{*A*, *C*, *G*}, {*A*, *G*, *H*}, {*C*, *G*, *H*}}. The expert sets {*A*, *C*} and {*E*, *F*} can not be linked because their union {*A*, *C*, *E*, *F*} is not a 3-*Eset*.

Pruning. *L*_*k*_ is a superset of $I_{k}^{E}$ and may contain some expert sets which can be excluded. We now introduce a definition that underlies the exclusion of such *k*-*Eset*. The minimum cost threshold is designated by *min*_*cost* which always records the cost of the current optimal team. The definition is as follows:

Definition 6 (Minimum Cost Threshold). Given a project *P*′ and a set of expert sets *L*_*k*_, the minimum cost threshold, if exists, is notated as *min*_*cost* representing the lowest cost of the expert set which can cover the project *P*′, i.e.,

$$\begin{array}{c} min\_cost=\min\{c(Eset)|Eset\in L_{k}\wedge s\left(P^{\prime }\right)\subseteq s\left(Eset\right)\}. \end{array}$$

Based on Definition 6, we present the following property.

Property 4. *Expert sets in L_k_ whose costs exceed the minimum cost threshold can be excluded from L_k_*.

In addition, assume there exist two expert sets *Eset*1, *Eset*2 ∈ *L*_*k*_, if *Eset*1 contains all the skills that *Eset*2 possesses and the cost of hiring *Eset*1 is less than hiring *Eset*2, we can replace *Eset*2 with *Eset*1 and remove *Eset*2 from *L*_*k*_. So we have the following property.

Property 5. *Given a set of expert sets L_k_*, ∀*Eset*1, *Eset*2 ∈ *L_k_, if Eset*1 *can cover Eset*2 *(i.e., s(Eset*2) ⊆ *s(Eset*1*)) and c(Eset*1) < *c(Eset*2*), we can exclude Eset*2 *from L_k_*.

We draw on both properties for pruning. If a *k*-*Eset* in *L*_*k*_ can be pruned, it must comply with the requirement of either of the two properties. In our running example, we assume *L*_1_ is {{*A*}, {*B*}, {*C*}, {*D*}, {*E*}, {*F*}, {*G*}, {*H*}} and *min*_*cost* is 8. We take the pruning step to discard 1-*Eset* {*B*} because the cost of {*B*} surpasses 8, i.e., *c*(*B*) = 10 > *min*_*cost*. On the other hand, we can delete 1-*Eset* {*A*} and {*E*} in the pruning step, owing to the fact that their skills can be covered by other 1-*Eset* whose costs are less than theirs. Here, they can entirely be substituted by another two 1-*Eset*s {*H*} and {*G*} respectively.

Algorithm 1 shows the pseudo-code of LPA. In Algorithm 1, first the projects P coalesce into one project *P*′ and we take a greedy strategy to work out a near-optimal solution (lines 2-8). The strategy greedily selects experts, one at a time, and assign it to the project *P*″, a duplicate of *P*′ (lines 4-5). Experts who can cover more skills of the project and incur lower cost are preferred. This greedy approach yields a current optimal team T and its corresponding *min*_*cost*. In lines 9-27, we search *L*_*k*_ for the *k*-*Eset*, which is returned as the output, that can handle *P*′ and carries the least cost. Firstly, *L*_1_ is initialized to a set of 1-*Eset*s, each of which is constituted by an expert in X (line 9). After that, we search *L*_1_ and update T and *min*_*cost* (lines 10-14), and by pruning, we get $I_{1}^{E}$ (line 15). For each *k* (*k* ≥ 2), the algorithm first generates *L*_*k*_ by linking $I_{k-1}^{E}$ and $I_{k-1}^{E}$ (line 17), and if *L*_*k*_ is empty, we will return the optimal team T and its corresponding *min*_*cost* (lines 18-20). Then, we try to identify the most optimal solution in current *L*_*k*_, if exists (lines 21-25). After that, it prunes the elements in *L*_*k*_ according to Property 4 and Property 5 (line 26). Consequently, $I_{k}^{E}$ which is used to be linked and generate *L*_*k*+1_ is formed.

**Algorithm 1** Linking-Pruning Algorithm.

**Require**: project set P, expert set X, cost function *c*, skill function *s*

**Ensure**: a team T and the corresponding cost *min*_*cost*

1: T←∅, *min*_*cost* ← 0

2: $P^{\prime }\leftarrow merge\left(P\right)$, *P*″ ← *P*′

3: **while** *s*(*P*″) ≠ ∅ **do**

4:  $x_{i}\leftarrow \arg\max_{x_{i}\in X\backslash T}\frac{|s\left(P^{\prime \prime }\right)\cap s\left(x_{i}\right)|}{c(x_{i})}$

5:  *s*(*P*″)←*s*(*P*″)∖*s*(*x*_*i*_)

6:  $T\leftarrow T\cup \{x_{i}\}$

7:  *min*_*cost* ← *min*_*cost* + *c*(*x*_*i*_)

8: **end while**

9: $L_{1}\leftarrow \{\left\{x_{i}\right\}|x_{i}\in X\}$

10: **for** *Eset* ∈ *L*_1_ **do**

11:  **if** *c*(*Eset*) < *min*_*cost* and *s*(*Eset*) ⊇ *s*(*P*′) **then**

12:   *min*_*cost* ← *c*(*Eset*), T←Eset

13:  **end if**

14: **end for**

15: $I_{1}^{E}\leftarrow L_{1}$        //Pruning

16: **for** (*k* ← 2;; *k*++) **do**

17:  $L_{k}\leftarrow I_{k-1}^{E}⋈I_{k-1}^{E}$  //Linking

18:  **if** *L*_*k*_ = ∅ **then**

19:   **return** T,*min*_*cost*

20:  **end if**

21:  **for** *Eset* ∈ *L*_*k*_ **do**

22:   **if** *c*(*Eset*) < *min*_*cost* and *s*(*Eset*) ⊇ *s*(*P*′) **then**

23:    *min*_*cost* ← *c*(*Eset*), T←Eset

24:   **end if**

25:  **end for**

26:  $I_{k}^{E}\leftarrow L_{k}$  //Pruning

27: **end for**

**Fig 4:**
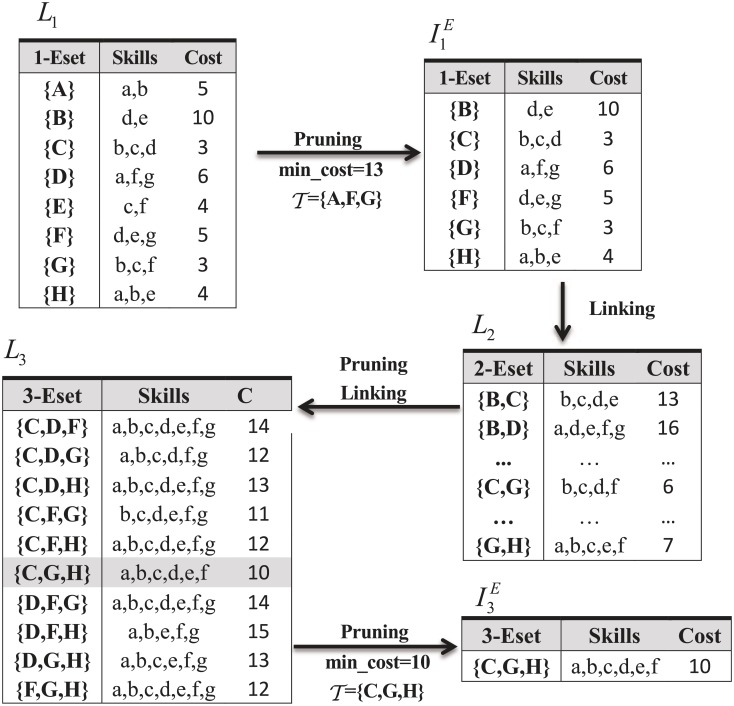
Procedure of LPA.

The procedure of LPA is well exemplified in Fig 4. In this example, experts and projects are apparently identical to those in Table 1 and we assume a project *P*′, which is merged by a set of projects P={1,2}, requires a set of skills *S*(*P*′) = {*a*, *b*, *c*, *d*, *e*, *f*}. Obviously, the participation constraint of each expert is beyond the total number of projects (i.e., 2). Our illustration focuses on the linking and pruning steps, and we assume a current optimal team T={A,F,G} and the corresponding *min*_*cost* 13 being reported by the greedy strategy of LPA (Note that for now, T is not the final outcome of the greedy strategy, and we choose this value simply for the sake of discussion).

From Fig 4 we observe that *L*_1_ is a set of 1-*Eset*s, each of which is composed of a single expert. By pruning, we can exclude {*A*} and {*E*} from *L*_1_ since they can entirely be replaced by {*H*} and {*G*} respectively (according to Property 5). After pruning, the 1-candidate expert set $I_{1}^{E}$ is formed, which can be seen from Fig 4. $I_{1}^{E}$ is employed for linking and generating *L*_2_. Similarly, by pruning and linking, we can generate *L*_3_ from *L*_2_. In *L*_3_, we obtain the current optimal team {*C*, *G*, *H*} which is preferable to {*A*, *F*, *G*}. Then, we update T={C,G,H} and *min*_*cost* = 10. Afterwards, we apply pruning and the 3-candidate expert set $I_{3}^{E}$ is formed. There exists only one 3-*Eset* {*C*, *G*, *H*} in $I_{3}^{E}$ suggesting that *L*_4_ can not be generated from only one 3-*Eset*. Therefore, we terminate the running process and return {*C*, *G*, *H*} as the best team T.

### Integer programming based algorithm

In essence, problem PFTH can be solved using integer programming. Let $A=\{a_{1},\cdots ,a_{k}\}$ denote the set of skills required by the projects in P without loss of generality. For any expert $x_{i}\in X$, we remove its skills not in A. Let *y*_*ij*_ indicate whether expert $x_{i}\in X$ apply skill $a_{j}\in A$ to the project set P. That is to say, *y*_*ij*_ = 1 if *x*_*i*_ participate in the project set P by his/her skill *a*_*j*_, 0 otherwise. As a result, PFTH can be formulated as

$$\begin{array}{cc} \text{min} & \sum_{i=1}^{n}\sum_{j=1}^{k}c\left(x_{i}\right)\times y_{ij}\\ s.t. & \sum_{i=1}^{n}\sum_{j=1}^{k}y_{ij}=k \end{array}.$$ (3)

## Algorithms for pcth

In this section we describe three algorithms for solving PCTH: the ProjectGreedy, the ExpertGreedy and the ExpertProjectGreedy. We now introduce them respectively. These algorithms can be applied to different problem instances. ProjectGreedy can yield a team with less cost and small size, and has relatively high time efficiency. ExpertGreedy can yield a team with high participation rate, i.e. the fraction of the total number of experts assigned to projects to the total cost of the team. ExpertProjectGreedy can yield a team with high skill utilization, i.e. the skills can be fully used. In Section, we will conduct experiments to show these advantages.

### The ProjectGreedy algorithm

The ProjectGreedy algorithm randomly picks projects, one at a time, which is then performed by the experts greedily selected by this technique. An expert *x*_*i*_ is assigned to the current project *p*_*j*_ if it maximizes:

$$\begin{array}{c} \frac{|s\left(p_{j}\right)\cap s\left(x_{i}\right)|\cdot w\left(x_{i}\right)}{c(x_{i})}, \end{array}$$ (4)

which shows an intuitive way to curtail the overall compensation. According to Eq 4, experts mastering more pertinent skills are preferred when they are vying for the same project. Meanwhile, to minimize the overall compensation, loose participation constraints and low costs are expected.

Having engaged in a project and still conforming to his participation constraint, the expert will be greedily assigned to other projects depending on the relevance of his skills (i.e., similarity of their skill sets). The similarity between two skill sets *s*′ and *s*″ is defined as follows:

$$\begin{array}{c} sim\left(s^{\prime },s^{\prime \prime }\right)=\frac{\left|s^{\prime }\cap s^{\prime \prime }\right|}{\left|s^{\prime }\cup s^{\prime \prime }\right|}. \end{array}$$ (5)

Therefore, given an expert *x*_*i*_, a project *p*_*j*_ is chosen to be covered such that it maximizes *sim*(*s*(*p*_*j*_), *s*(*x*_*i*_)). The pseudo-code of ProjectGreedy is listed in Algorithm 2.

**Algorithm 2** Pseudo-code of ProjectGreedy.

**Require**: expert set X, project set P, participation constraint function *w*, cost function *c*, skill function *s*

**Ensure**: a team T and the corresponding cost c(T)

1: T←∅

2: **while** P≠∅ **do**

3:  $p_{j}\leftarrow randomselect\left(P\right)$

4:  **while** *s*(*p*_*j*_) ≠ ∅ **do**

5:   $x_{i}\leftarrow \arg\max_{x_{i}\in X}\frac{|s\left(p_{j}\right)\cap s\left(x_{i}\right)|\cdot w\left(x_{i}\right)}{c(x_{i})}$

6:   **for** *t* ← 1 to *w*(*x*_*i*_) − 1 **do**

7:    $p^{\prime }\leftarrow \arg\max_{p^{\prime }\in P\backslash \left\{p_{j}\right\}}sim\left(s\left(p^{\prime }\right),s\left(x_{i}\right)\right)$

8:    *s*(*p*′)←*s*(*p*′)∖*s*(*x*_*i*_)

9:   **end for**

10:   $X\leftarrow X\backslash \{x_{i}\}$

11:   *s*(*p*_*j*_)←*s*(*p*_*j*_)∖*s*(*x*_*i*_)

12:   $T\leftarrow T\cup \{x_{i}\}$

13:  **end while**

14:  $P\leftarrow P\backslash \{p_{j}\}$

15: **end while**

16: **return** T,c(T)

T is initialized to an empty set at the very beginning. After that, we start the iterative process on the space of projects. In each iteration, we first randomly select a project *p*_*j*_ from P (line 3), and then we greedily choose experts, one at a time, to perform *p*_*j*_ (line 5). The process does not cease until *p*_*j*_ is totally covered (lines 4-13). Afterwards, we exclude *p*_*j*_ from the project set P (line 14). To put it differently, an expert who has joined a project *p*_*j*_ will be continually and greedily assigned to other projects on condition that he still complies with his participation constraint (lines 6-9).

According to Table 3, the size of P is *m* (line 2), so the algorithm will iterate at most *m* times through the space of P. For each project, we assume the average number of skills of it is *q* (line 4), so is the maximum amount of iteration through lines 4-13 for our algorithm. In each iteration, it takes *n* times to select an ideal expert (line 5), and at most *w* * *m* (*w* denotes the average number of projects in which an expert can participate simultaneously) times to assign the expert to other projects (lines 6-9). Therefore, the worst-case running time of ProjectGreedy adds up to *O*(*mq*(*n* + *wm*)), i.e., *O*(*mqn* + *wm*^2^*q*).

### The ExpertGreedy algorithm

The ExpertGreedy algorithm greedily picks experts, one at a time, and then it greedily assigns the expert to projects. The expert is chosen from the expert set such that it maximize:

$$\begin{array}{c} \frac{|ss\cap s(x_{i})|\cdot w\left(x_{i}\right)}{c(x_{i})}, \end{array}$$ (6)

where $ss\leftarrow \cup_{p_{j}\in P}s\left(p_{j}\right)$ denotes the union of skills of all the remaining projects. When choosing experts, ExpertGreedy perceives all the remaining projects as a whole and experts who cover more skills of the whole are preferred. Moreover, to minimize the compensation, the method favors experts with loose participation constraints and low costs. Once an expert has been chosen by the algorithm, he will be greedily assigned to projects according to the similarity of their skills. The pseudo-code of ExpertGreedy is listed in Algorithm 3.

**Algorithm 3** pseudo-code of ExpertGreedy.

**Require**: expert set X, project set P, participation constraint function *w*, cost function *c*, skill function *s*

**Ensure**: a team T and the corresponding cost c(T)

1: T←∅

2: $ss\leftarrow \cup_{p_{j}\in P}s\left(p_{j}\right)$

3: **while** *true* **do**

4:  $x_{i}\leftarrow \arg\max_{x_{i}\in X}\frac{|ss\cap s(x_{i})|\cdot w\left(x_{i}\right)}{c(x_{i})}$

5:  **for** *t* ← 1 to *w*(*x*_*i*_) **do**

6:   $p_{j}\leftarrow \arg\max_{p_{j}\in P}sim\left(s\left(x_{i}\right),s\left(p_{j}\right)\right)$

7:   *s*(*p*_*j*_)←*s*(*p*_*j*_)∖*s*(*x*_*i*_)

8:   **if** *s*(*p*_*j*_) = ∅ **then**

9:    $P\leftarrow P\backslash \{p_{j}\}$

10:   **end if**

11:  **end for**

12:  $T\leftarrow T\cup \{x_{i}\}$

13:  $ss\leftarrow \cup_{p_{j}\in P}s\left(p_{j}\right)$

14:  **if** *ss* = ∅ **then**

15:   **return** T,c(T)

16:  **end if**

17:  $X\leftarrow X\backslash \{x_{i}\}$

18: **end while**

We also start with an empty T set and initialize set *ss* to the union of skills of all the projects (lines 1-2). Then, the algorithm keeps iterating until *ss* becomes empty (lines 3-18). In each iteration, we opt for the expert *x*_*i*_ who maximizes Eq 6 (line 4). After that, *x*_*i*_ is greedily assigned to projects with the relevance of his skills being the chief determinant (lines 5-11). Moreover, *ss* gradually shrinks to an empty set as the project assignment progresses. If so, we return the team T and the corresponding cost and terminate the algorithm (lines 14-16).

In ExpertGreedy, the size of *ss* is *k* (number of skills), so it iterates at most *k* * *m* times through lines 3-18 (assume each chosen expert can cover only one skill of one project). Then, it iterates *n* times in line 4 and *w* * *m* (*w* denotes the average number of projects in which an expert can participate simultaneously) times through lines 5-11. Therefore, the worst-case running time of ExpertGreedy amounts to *O*(*km*(*n* + *wm*)), i.e., *O*(*kmn* + *km*^2^*w*).

### The ExpertProjectGreedy algorithm

**Algorithm 4** pseudo-code of ExpertProjectGreedy.

**Require**: expert set X, project set P, participation constraint function *w*, cost function *c*, skill function *s*

**Ensure**: a team T and the corresponding cost c(T)

1: T←∅

2: U←X×P

3: *c*_*temp*_ ← *c*

4: **while** *U* ≠ ∅ **do**

5:  $\left(x_{i},p_{j}\right)\leftarrow \arg\max_{(x_{i},p_{j})\in U}\frac{|s\left(x_{i}\right)\cap s\left(p_{j}\right)|}{c_{temp}\left(x_{i}\right)}$

6:  *U* ← *U*∖{(*x*_*i*_, *p*_*j*_)}

7:  *c*_*temp*_(*x*_*i*_) ← 1

8:  *s*(*p*_*j*_)←*s*(*p*_*j*_)∖*s*(*x*_*i*_)

9:  *w*(*x*_*i*_)←*w*(*x*_*i*_) − 1

10:  **if** *s*(*p*_*j*_) = ∅ **then**

11:   remove each (⋅, *p*_*j*_)∈*U*

12:  **end if**

13:  **if** *w*(*x*_*i*_) = 0 **then**

14:   remove each (*x*_*i*_, ⋅)∈*U*

15:  **end if**

16:  $T\leftarrow T\cup \{x_{i}\}$

17: **end while**

18: **return** T,c(T)

ProjectGreedy and ExpertGreedy start with project selection and expert selection respectively. Unlike the two algorithms, in this section, we propose another alternative dubbed ExpertProjectGreedy which combines an expert and a project into a match pair and perceives them as a whole. A match pair (*x*_*i*_, *p*_*j*_) represents that an expert *x*_*i*_ is assigned to a project *p*_*j*_. We also define the marginal gain of assignment (*x*_*i*_, *p*_*j*_) as $\frac{|s\left(x_{i}\right)\cap s\left(p_{j}\right)|}{c(x_{i})}$. Initially possible match pairs totaling |X×P| constitute a set *U*. During the execution, the algorithm greedily picks match pairs from *U*, one at a time, such that it maximizes the marginal gain. When a match pair (*x*_*i*_, *p*_*j*_) is picked by our algorithm, the expert *x*_*i*_ is assigned to the project *p*_*j*_, and we update the status of *x*_*i*_ (*w*(*x*_*i*_) = *w*(*x*_*i*_) − 1) and *p*_*j*_ (*s*(*p*_*j*_) = *s*(*p*_*j*_)∖*s*(*x*_*i*_)). If an expert *x*_*i*_ is not available ((*w*(*x*_*i*_) = 0)) any more, we will remove all the match pairs involving *x*_*i*_ (*x*_*i*_, ⋅) from *U*. By the same token, if a project *p*_*j*_ is totally covered, we too will remove all the match pairs involving *p*_*j*_ (⋅, *p*_*j*_) from *U*. The pseudo-code of ExpertProjectGreedy is displayed in Algorithm 4.

First we initialize T to an empty set and the set of potential match pairs to X×P, with *c*_*temp*_ being a duplicate of *c*. The match pair yielding the largest marginal gain is picked in line 5. After a match pair (*x*_*i*_, *p*_*j*_) has been chosen, we exclude it from *U* and update the status of *x*_*i*_ and *p*_*j*_ (lines 7-9). The reason for setting *c*_*temp*_(*x*_*i*_) = 1 is that the cost of a selected expert is only considered at most once in the entire process. The algorithm retains the possible matches in *U* in lines 10-15. It can be observed that ExpertProjectGreedy iterates at most |X×P| times to attain an ideal team, but in practice, the value can be much smaller.

The while-loop iterates at most *m* * *q* (*q* counts the average number of skills of each project) times (although the size of *U* is *n* * *m*, the while-loop will be halted once all the projects have been performed). Within each iteration of the while-loop, it takes at most *n* * *m* times to pick a match pair reaping the most benefit (line 5). So ExpertProjectGreedy gives the worst-case running time *O*(*mq*(*n* * *m*)), i.e., *O*(*m*^2^ *nq*).

## Experiments

In this section, we evaluate the performance of the proposed algorithms through experiments. Our algorithms are implemented using Java. All the experiments are conducted on a PC with Intel(R) Core(TM) 2.94GHz CPU and 2.0GB memory.

### Datasets

Our experiments are performed on both real and synthetic datasets. The real datasets are collected from two large labor markets: freelancer.com and guru.com, which we refer to as Freelancer and Guru respectively. On both websites, employers post projects with the required skills that they are avidly seeking. Experts with different skillsets and salary demands apply for one or more projects, and are evaluated by the employers. Besides, for each expert, we impose a participation constraint on him which restricts the maximum number of projects he can enter in parallel. We randomly generate this constraint ranging from 1 to 3 for all experts. Additionally, the synthetic data which is named SynData is also produced in a random manner. Summary statistics from these datasets are exhibited in Table 4. In Table 4, |P|, |X| and |A| count the number of projects, the number of experts and the number of skills respectively. For example, we glean information on 6363 experts and 1239 projects which embody 592 skills for Guru dataset. $\bar{\left|s(p_{j})\right|}$, $\bar{\left|s(x_{i})\right|}$ and $\bar{\left|c(x_{i})\right|}$ stand for the average number of skills per project, the average number of skills per expert and the average cost per expert respectively. The maximum/minimum number of skills regarding all projects is denoted by |*s*(*p*_*j*_)|_*max*_ and |*s*(*p*_*j*_)|_*min*_. Analogous to the treatment for projects, |*s*(*x*_*i*_)|_*max*_ and |*s*(*x*_*i*_)|_*min*_ represent the maximum/minimum number of skills concerning all the experts.

**Table 4:** Summary statistics from datasets.

Statistics	Guru	Freelancer	SynData
|P|	1239	464	500
|X|	6363	1147	1500
A	592	55	100
$\bar{s(p_{j})}$	4.098	3.254	17.518
|*s*(*p*_*j*_)|_*max*_	28	5	30
|*s*(*p*_*j*_)|_*min*_	1	1	5
$\bar{s(x_{i})}$	9.606	4.59	3
|*s*(*x*_*i*_)|_*max*_	104	5	5
|*s*(*x*_*i*_)|_*min*_	1	1	1
$\bar{\left|c(x_{i})\right|}$	37.891	19.609	12.598

### Performance evaluation for pfth

**Fig 5:**
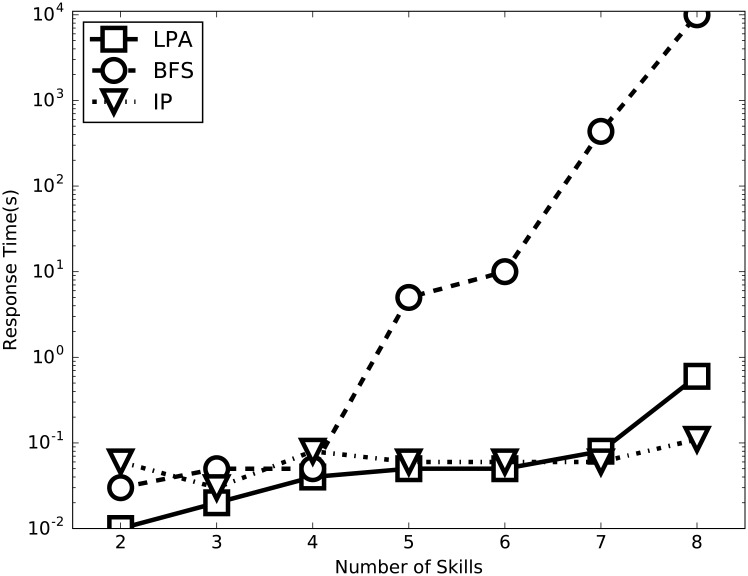
Performance evaluation of LPA and BFS with respect to |*s*(*P*′)|.

**Fig 6:**
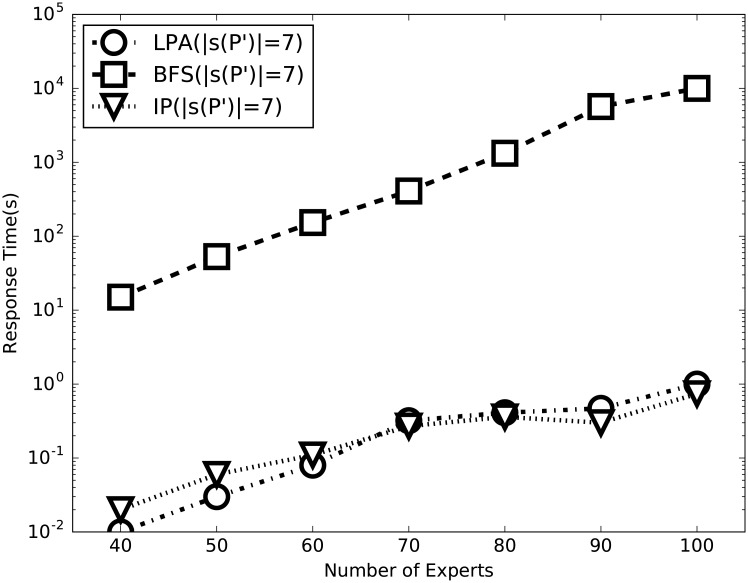
Performance evaluation of LPA and BFS with respect to |*X*|.

In this section, we evaluate the efficiency of the integer programming compared with the Linking-Pruning algorithm (LPA) and the Brute Force Search (BFS). We draw on the knowledge from Section that LPA is particularly sensitive to the number of skills of the merged project and the number of experts. Hence, the effect of |*s*(*P*′)| (i.e., the number of skills of the merged project *P*′) and |X| (i.e., the number of experts) are assessed in this section. Since the scale of project differs enormously between the two real-world datasets, too small in Freelancer (each project asks for at most 5 skills) and Guru too large, we opt for SynData as our experimental data. In each experiment, we randomly select projects and merge them into a larger one *P*′ and our evaluation focuses on *P*′. Then, we report the results which are plotted in Figs 5 and 6.

Fig 5 shows the corresponding response time when the number of skills required by the project varies. The number of experts is set to 100 (i.e., |X|=100). Obviously, LPA greatly outperforms BFS. And LPA slightly outperforms integer programming (abbreviated as IP in the figure) when the number of skills is small. From Fig 6 we also notice that LPA tends to be less radically affected by |*s*(*P*′)| than BFS due to the effectiveness of the pruning strategies, and IP could be scarcely influenced by the number of skills.

We alter the number of experts to compare their response time in Fig 6. Three different experimental setups are in place, |*s*(*P*′)| = 7 for BFS and |*s*(*P*′)| = 7 for LPA, and |*s*(*P*′)| = 7 for integer programming (abbreviated as IP in the figure). From this figure we can see that LPA and IP are distinctly superior to BFS in every experimental setup. And LPA has the comparable time efficiency to IP when the number of skills is small. Moreover, LPA and IP are far less sensitive to |X| than BFS.

From the preceding comparison we can reach the conclusion that IP and LPA significantly outperforms BFS, in terms of both the runtime and the scale of the merged project and experts. When the number of skills is small, LPA have the comparable time efficiency to IP.

### Performance evaluation for pcth

In this section, we evaluate the algorithms proposed for PCTH. To this end, we report the overall cost, team size, skill utilization, participation rate and response time achieved by each algorithm respectively, by providing them with different amounts of projects, i.e., |P|∈{10,20,30,40,50}. Apart for the three algorithms described in Section, we also employ a naive greedy heuristics algorithm called RandomExpert as an additional baseline. RandomExpert randomly selects experts, one at a time, from the space of expert set. Then it greedily assigns the expert to the projects based on the similarity of skills. The algorithm does not cease selecting experts until all the projects have been fully covered.

We carried out our experiments on three datasets: SynData, Guru and Freelancer. In each experiment, we compare the performance of the proposed algorithms. The projects are selected randomly, and for each evaluation our experiments are repeated 100 times with the average results being reported.

#### Cost evaluation

**Fig 7:**
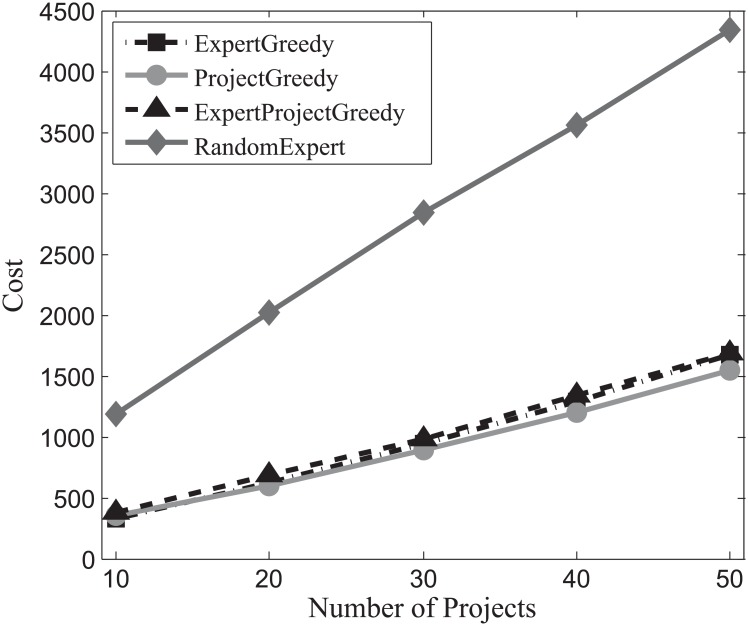
The evaluation of the achieved costs on SynData.

**Fig 8:**
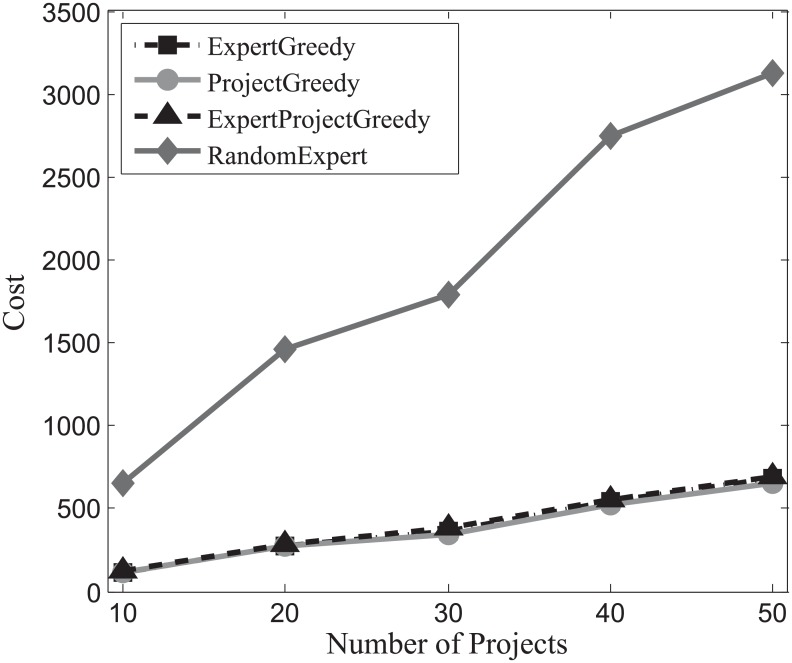
The evaluation of the achieved costs on Guru.

**Fig 9:**
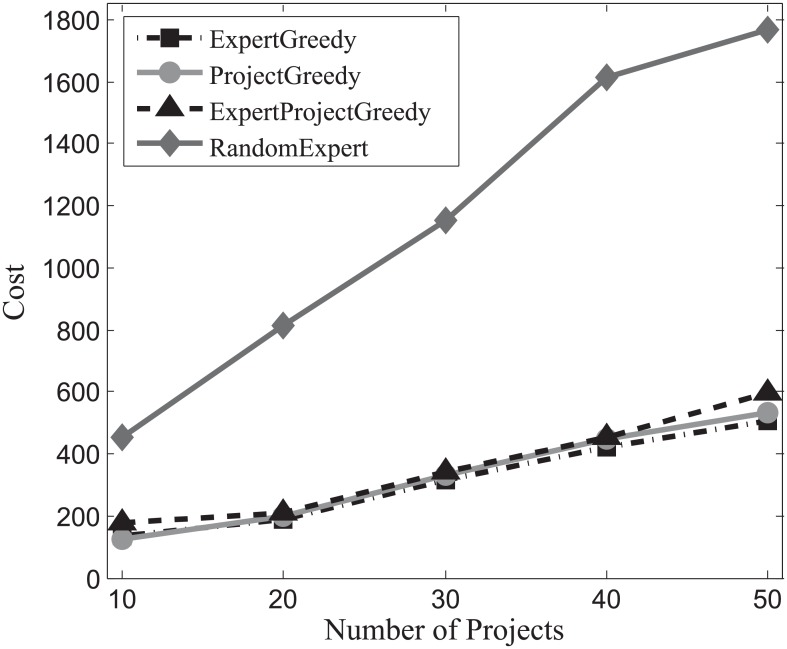
The evaluation of the achieved costs on Freelancer.

First we assess the cost of the team incurred by each algorithm on the three datasets. With the increase of the number of projects, the carried costs on SynData, Guru and Freelancer are ploted respectively in Figs 7, 8 and 9. It can be observed that with the increase of the number of projects, the associated costs of all the algorithms also escalate. All the algorithms except RandomExpert perform comparably which implies that there exist a multitude of skilled and cost-effective experts who can accomplish the required projects in each dataset. Therefore, no matter we concentrate on the projects (ProjectGreedy), the experts (ExpertGreedy) or both (ExpertProjectGreedy), the outcomes seem remarkably alike. RandomExpert bears higher cost than others because it disregards the expenses claimed by the experts. Additionally, from Table 4 we can find that the average number of skills per project of SynData vastly exceeds the other two datasets’ while the average number of skills per expert of SynData is below its two counterparts’. Therefore, even though the average cost per expert of SynData is lower than the other two datasets’, the total costs of SynData far surpass the other two datasets’.

Generally, we can draw the conclusion that when involving compensation costs, the three proposed algorithms behave similarly and are superior to the baseline.

#### Team size evaluation

**Fig 10:**
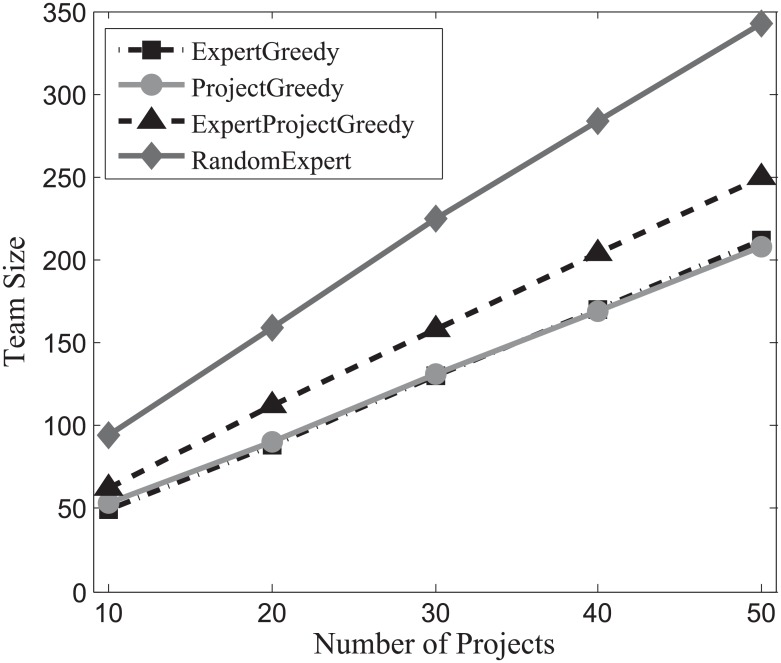
The evaluation of team size on SynData.

**Fig 11:**
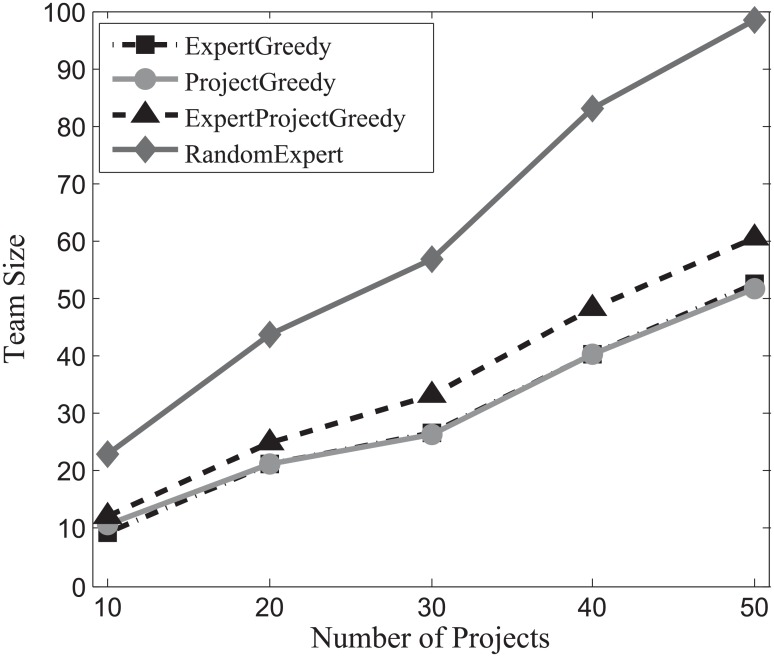
The evaluation of team size on Guru.

**Fig 12:**
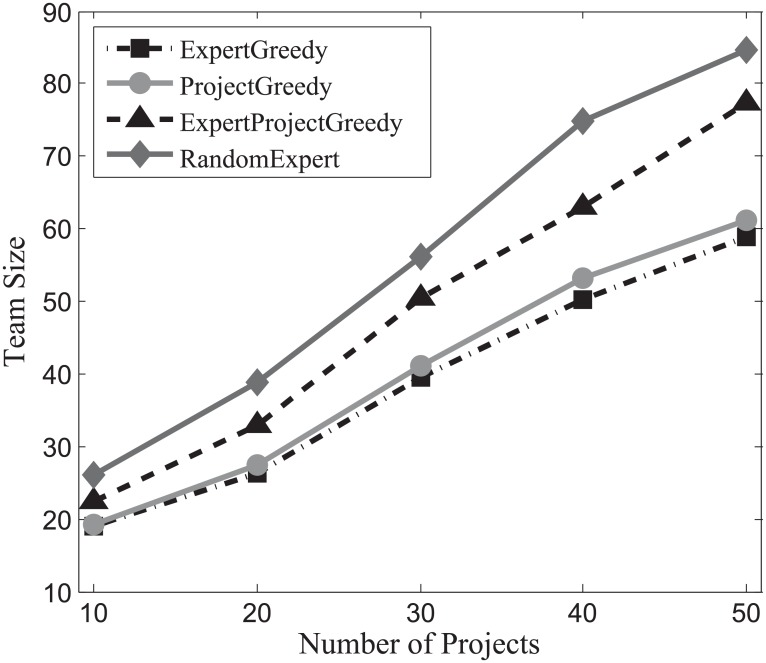
The evaluation of team size on Freelancer.

The success of a project hinges not only on the expertise of the individuals, but also on how effectively they communicate with each other [14]. Generally speaking, the larger the team size, the harder for experts communicate with each other. Therefore, team size occupies a vital role in the success of a project. In this section, we gauge the impact of team size on our algorithms and the results on SynData, Guru and Freelancer are shown in Figs 10, 11 and 12 respectively.

As can be seen in these figures, RandomExpert are prone to assemble large teams, followed by the ExpertProjectGreedy. This can be interpreted as: when deciding on an expert, RandomExpert randomly selects an expert, and overlooks the coverage of skills and the number of projects that the expert can be assigned to simultaneously, which in turn gives rise to large teams for the projects. ExpertProjectGreedy considers the similarity between the skills of experts and projects. However, many experts in the team created by ExpertProjectGreedy may not be fully exploited, i.e., the number of projects which an expert engages in falls below his participation constraint. On the other hand, ExpertGreedy and ProjectGreedy not only take into account the skill relevance of an expert and his participation constraint but also are directed at harnessing the capabilities of experts to the fullest. That is why the team size of ExpertGreedy and ProjectGreedy are smaller than the other two algorithms’. For comparison, the size of projects of SynData is deliberately set to top the other two datasets’. It can be observed that SynData yields the largest team size regardless of other factors including the number of projects, the size of projects or the type of running algorithm on the three datasets.

Generally, we can conclude that the team size of ExpertGreedy and ProjectGreedy are smaller compared with their peers.

#### Skill utilization evaluation

Then we analyze skill utilization of the proposed algorithms. Given a project set P and a team of experts T that can perform the projects, the skill utilization *ψ* is defined as follows:

$$\begin{array}{c} \psi =\frac{\sum_{p_{j}\in P}\left|s(p_{j})\right|}{\sum_{x_{i}\in T}|s\left(x_{i}\right)|\cdot w\left(x_{i}\right)}, \end{array}$$ (7)

where the numerator returns the number of skills required by projects (i.e., the number of skills experts utilized), and the denominator denotes the sum of the number of experts’ skills, with the participation constraint being considered. Obviously, it reflects the ratio of skill utilization.

**Fig 13:**
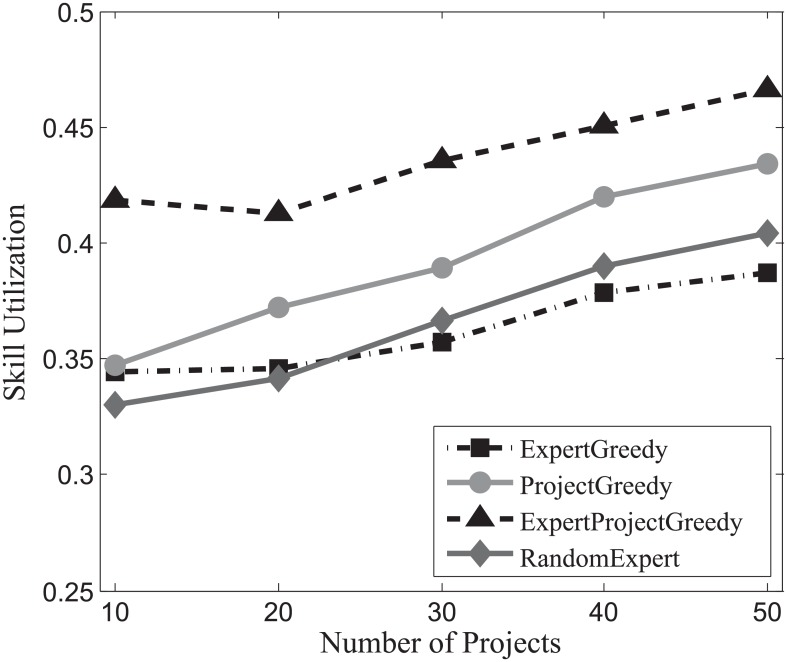
The evaluation of the skill utilization on SynData.

**Fig 14:**
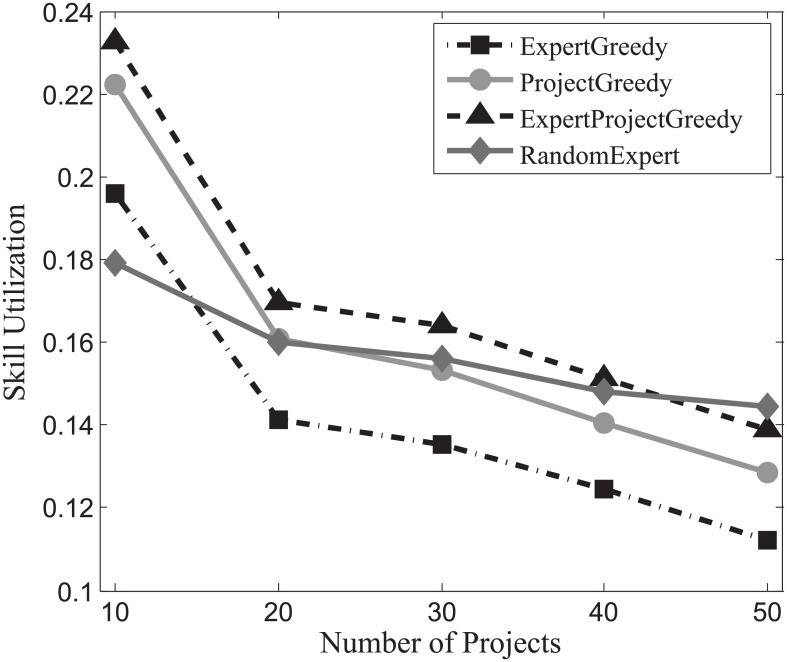
The evaluation of the skill utilization on Guru.

**Fig 15:**
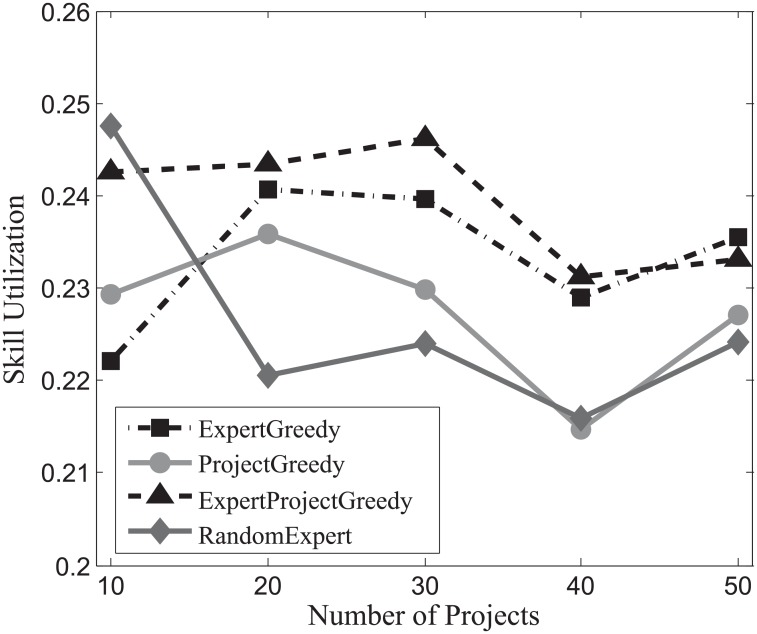
The evaluation of the skill utilization on Freelancer.

The results attained by each algorithm on SynData, Guru and Freelancer are shown in Figs 13, 14 and 15 respectively. From the figures, it is noteworthy that ExpertProjectGreedy fares much better than the others regarding skill utilization. In fact, ExpertProjectGreedy accomplishes this through two measures. First, it greedily selects expert-project match pairs with respect to their similarity of skills, which manages to exploit the skills of the experts to the most possible extent. Second, after a match pair (*x*_*i*_, *p*_*j*_) has been selected, the cost of *x*_*i*_ plunges to 1 (see Algorithm 4) indicating a strong likelihood that the expert will be chosen later.

Note that skill utilization declines with the increase of the number of projects for Guru, which contrasts starkly with the other two datasets. Recall that in Table 4 the average number of skills of expert more than doubles that of project for Guru, suggesting that the increase of team members can amplify the effect that more irrelevant skills reduce the ratio of utilization. As was discussed earlier, team size is determined by both the number of projects and the skills of experts. Thus, it makes sense to see that the ratio of skill utilization drops with the increase of the quantity of projects for Guru.

On the whole, the skill utilization attained by ExpertProjectGreedy is superior to the others’.

#### Participation rate evaluation

Since the participation constraint of experts is an essential condition in our problem, we examine the participation rate of the teams formed by all the algorithms. The participation rate *β* is defined as follows:

$$\begin{array}{c} \beta =\frac{\sum_{p_{j}\in P}|com(p_{j})|}{\sum_{x_{i}\in T}w(x_{i})}. \end{array}$$ (8)

The denominator of the fraction represents the sum of *w*(*x*_*i*_) of each team members *x*_*i*_, and the numerator gives the sum of the number of projects each team member is involved in. Obviously, the value of this fraction ranges from 0 to 1. The number of projects that experts engage in is maximized when the participation rate *β* reaches 1. Conversely, *β* = 0 indicates that no expert is assigned to any project.

**Fig 16:**
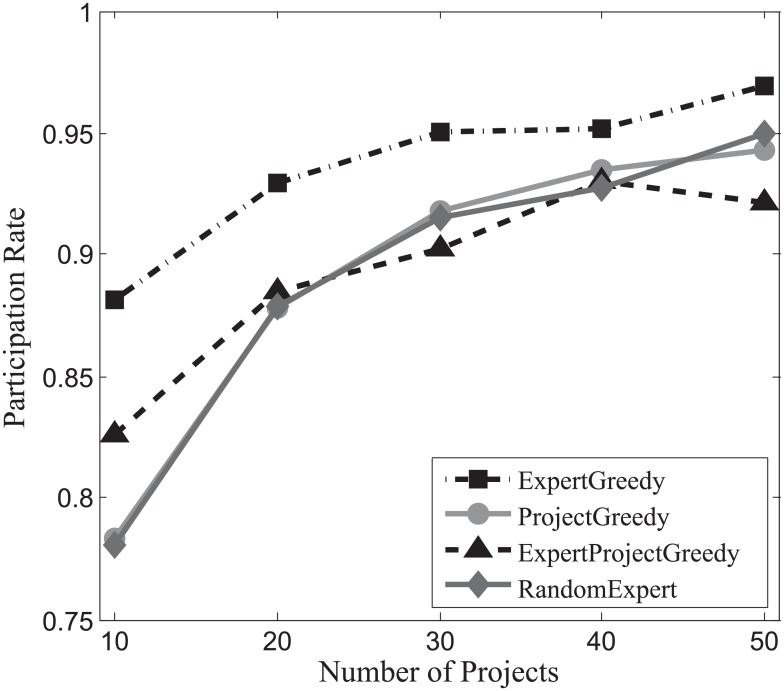
The evaluation of participation rate on SynData.

**Fig 17:**
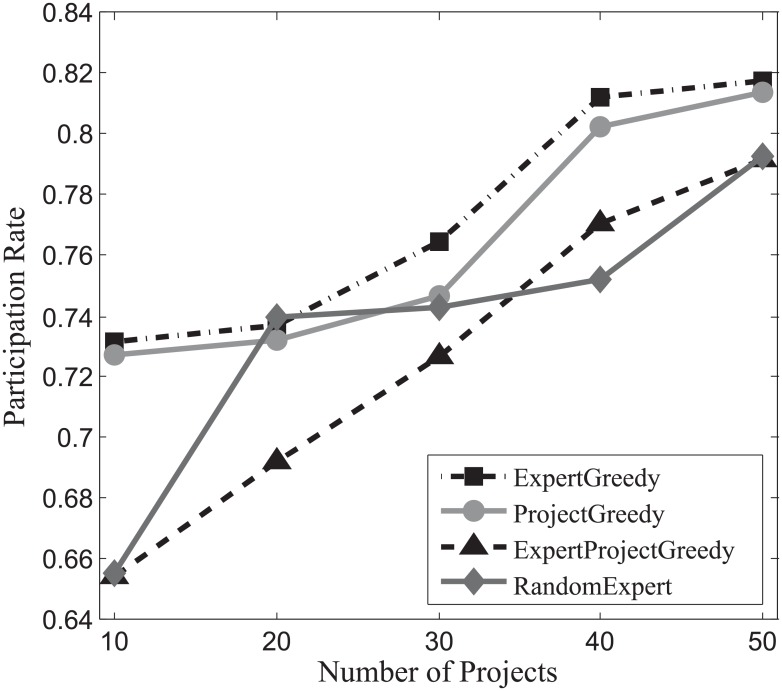
The evaluation of participation rate on Guru.

**Fig 18:**
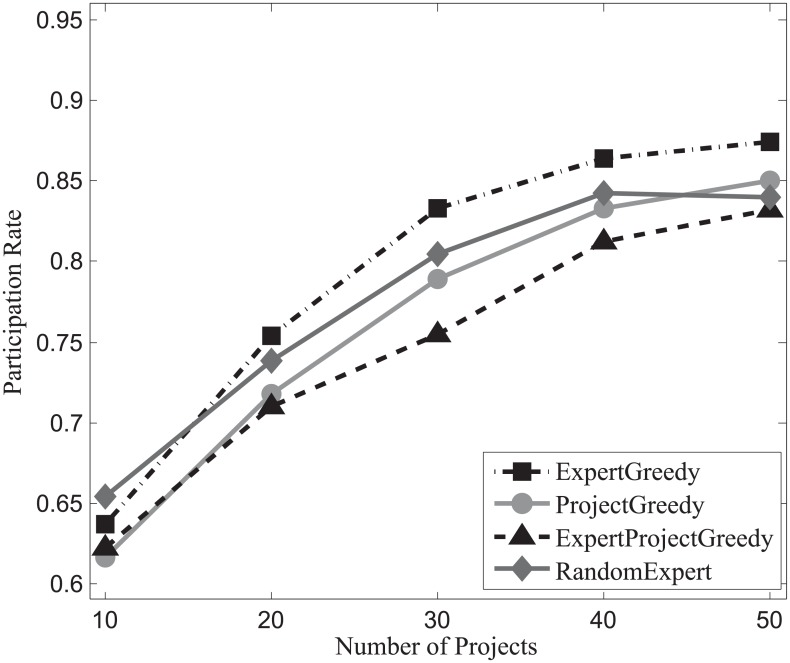
The evaluation of participation rate on Freelancer.

Figs 16, 17 and 18 depict the participation rates characterizing four algorithms on SynData, Guru and Freelancer. A consistent trend emerges followed by all the algorithms on different datasets that positive correlation between the participation rate *β* and the amount of projects can be identified. This can be primarily ascribed to the fact that more projects bring about larger skill sets which allow for more possibilities for experts to take on different jobs in parallel. From these figures, we can also observe that ExpertGreedy and ProjectGreedy all perform better than ExpertProjectGreedy because they employ fairly distinctive greedy strategies. In ExpertGreedy, experts are assigned to projects before his participation constraint falls to zero or simply he cannot fulfill the duties of the remaining projects. Similarly in ProjectGreedy, an expert can still engage in other projects after he has already been selected for one job, provided his participation constraint will not be violated. ExpertProjectGreedy which merely concentrates on the best match pair in every iteration differs substantially from the preceding two alternatives. Furthermore, ExpertGreedy holds a narrow lead over ProjectGreedy, which can be ascribed to the fact that in every iteration we always opt for the expert with the most pertinent skills so he in turn will be more inclined to join in other projects at the same time. Although ProjectGreedy approaches this problem from the perspective of one specific project, the skillset of a chosen expert still bears the strongest resemblance to that of the project.

Hence we can arrive at the conclusion that ExpertGreedy surpasses the other 3 algorithms in terms of participation rate.

#### Response time evaluation

**Fig 19:**
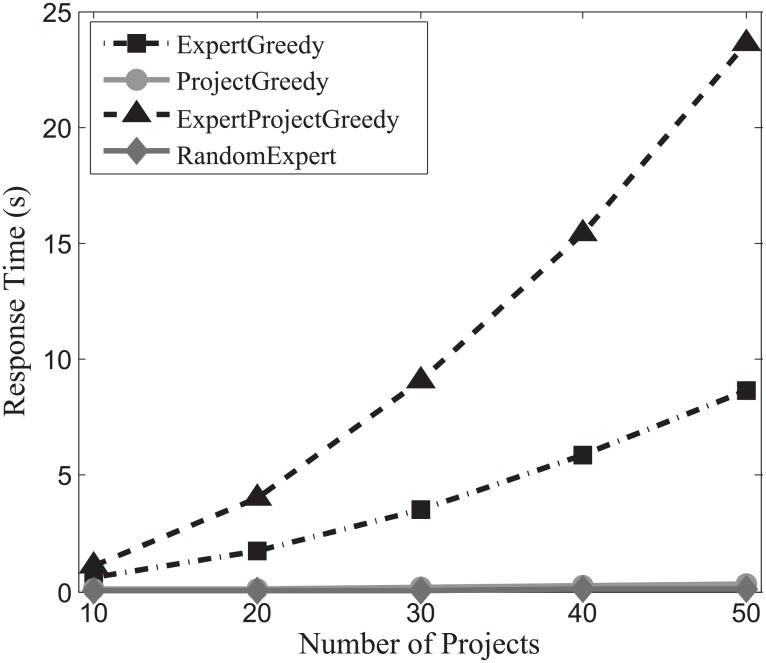
The evaluation of response time on SynData.

**Fig 20:**
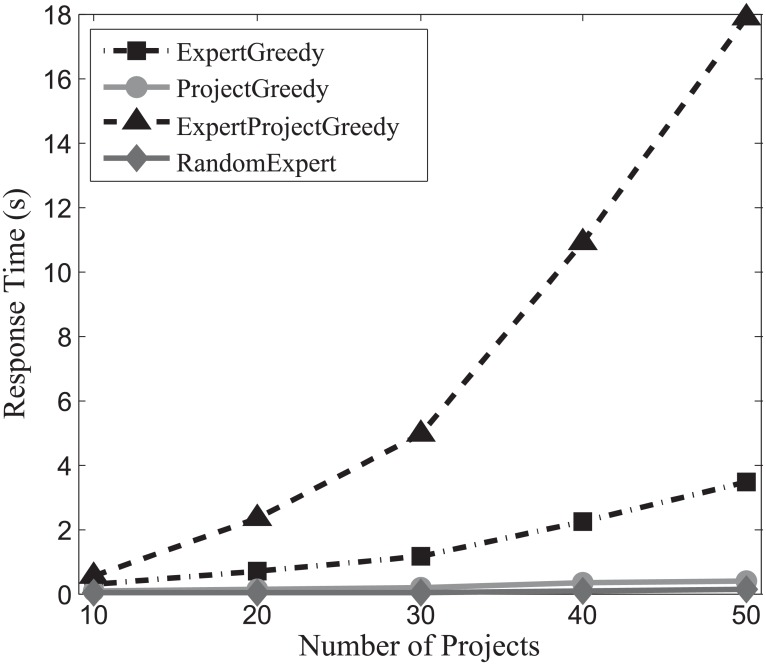
The evaluation of response time on Guru.

**Fig 21:**
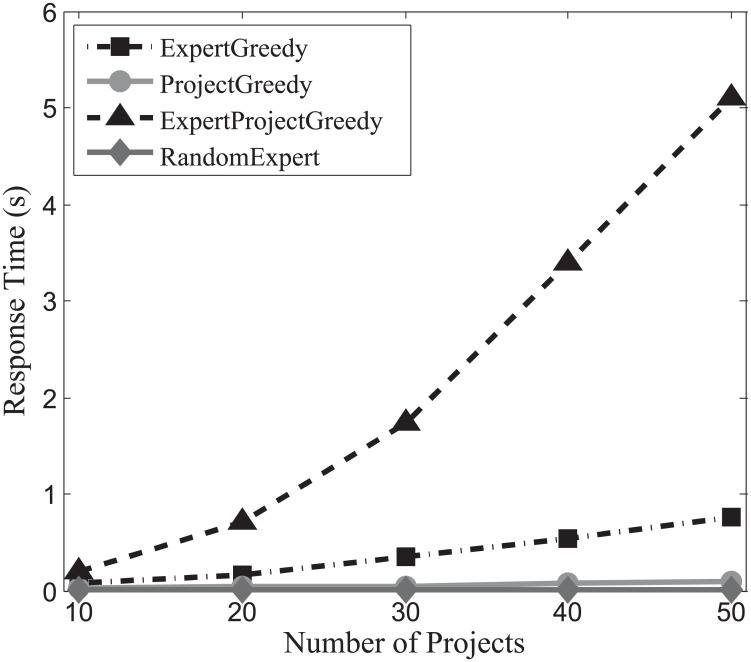
The evaluation of response time Freelancer.

In order to investigate the efficiency of the algorithms, we continue to conduct experiments on the three datasets, and the experimental results are displayed in Figs 19, 20 and 21. From these figures, we can observe that the response time of both ProjectGreedy and RandomExpert barely rises, vastly outperforming the other two from beginning to end. This can be explained by the fact that the two algorithms iterate fewer times on the space of expert set than ExpertGreedy or ExpertProjectGreedy does. In all the three datasets, the number of experts considerably surpasses that of projects. Therefore, iterating too many times on the space of experts will consume more time. Specifically, ProjectGreedy tends to select experts in a way that each project can be performed one by one. With the iteration progressing, the number of the remaining projects drops fast, and as a consequence, so does that of iteration on the space of experts. This differentiates ProjectGreedy from ExpertGreedy which treats the projects as a whole when deciding on an expert. Therefore, ProjectGreedy outperforms ExpertGreedy regarding response time under the same circumstances. Additionally, ExpertProjectGreedy iterates too many times on the space of X×P which apparently entails more time than the others. For this reason, as can be observed from the figures, ExpertProjectGreedy manifests greater susceptibility to the number of projects than the others.

Generally speaking, RandomExpert and ProjectGreedy are the most efficient algorithms among the four.

## Conclusions

In this paper, we proposed formalizations and algorithmic solutions for the participation constrained team hire problem (PCTH), where the goal is to hire a team of participation-constrained experts to complete all given projects such that the total cost is minimized. This is the first work to investigate the participation constrained team hire problem. We studied a special case of PCTH and introduced an efficient algorithm that identifies an exact solution for it. For the general PCTH, we proved that it is NP-hard and presented three algorithms. In a thorough experimental evaluation, we appraised the performance of our algorithms, and compared them with reasonable baseline approaches. We conclude that our algorithms on both synthetic and real datasets outperform the baseline algorithms significantly. In the future, we will embark on exploring how the preferences of experts regarding projects can shape this issue. That is, we would like to consider the scenario when an expert explicitly expresses his intense interest for a particular project, which will certainly serve as a vital factor in assigning experts to jobs.

## Supporting information

S1 Dataset Freelancer. (ZIP)

S2 Dataset Guru. (ZIP)

S3 Dataset SynData. (ZIP)
